# Contextualising Experiences of Co-Occurring Mental Ill-Health and Substance Use Among Trans, Non-Binary, and Gender Diverse Young People: Implications for Tailored Harm Reduction Approaches

**DOI:** 10.1007/s10597-024-01342-y

**Published:** 2024-09-02

**Authors:** Sasha Bailey, Ashleigh Lin, Angus Cook, Sam Winter, Vanessa Watson, Dani Wright Toussaint, Emma L. Barrett, Nicola C. Newton, Yael Perry, Lucinda Grummitt, Penelope Strauss

**Affiliations:** 1 Faculty of Medicine and Health, The Matilda Centre for Research in Mental Health and Substance Use, The University of Sydney, Level 6 G05 Jane Foss Russell Building, Camperdown, NSW 2050 Australia; 2 School of Population and Global Health, University of Western Australia, Perth, Australia; 3 School of Population Health, Curtin University, Bently, Australia; 4 Western Australian Department of Health, YouthLink, North Metropolitan Area Health Service, Nedlands, Australia; 5 Telethon Kids Institute, University of Western Australia, Perth, Australia

**Keywords:** Trans, Non-binary, Gender diverse, Mental ill-health, Substance use, Harm reduction

## Abstract

Though significant research highlights higher rates of mental ill-health and substance use among trans, non-binary and gender diverse (henceforth ‘trans’) young people, little research has considered patterns, contextual characteristics, and correlates of co-occurring experiences of mental ill-health and substance use among trans young people. Using data from the Trans Pathways study, we used prevalence ratios and age- and gender-adjusted logistic regression models to examine prevalence and differences of co-occurring substance use (past six-month cigarette use, alcohol use, and other drug use) and contextual characteristics of substance use (past six-month solitary alcohol and/or drug use, substance use for coping) by mental ill-health (depression disorder, anxiety disorder, past 12-month self-harm thoughts and behaviours, suicidal thoughts, planning, and attempt/s). Age- and gender-adjusted models assessed associations between co-occurring depressive and anxiety disorders and recent cigarette, alcohol, and other drug use (six co-occurring items total) and 18 interpersonal stressors. Significantly increased odds of smoking or recent use of cannabis or sedatives was observed among trans young people reporting depressive disorder, anxiety disorder (aORs ranging 1.8–3.1). Trans young people who reported recent smoking or use of cannabis, inhalants, or sedatives, had 40% to 80% reduced odds of past 12-month self-harm thoughts, self-harm behaviours, suicidal thoughts, and suicide attempt/s (aORs ranging 0.2–0.6). On the other hand, solitary alcohol and/or other drug use and substance use for coping was significantly associated with increased odds of all mental ill-health outcomes. Issues with school, secure housing, and intimate partner abuse were the most robust correlates of co-occurring mental ill-health and substance use. Trans young people using substances, especially cigarettes, cannabis, and sedatives, often so do with co-occurring experiences of depression and anxiety though limited substance use in more ‘social’ contexts may confer benefits for preventing self-harm and suicide thoughts and behaviours. Continued research in partnership with trans young people is warranted to conceptualise more nuanced and precise conceptual parameters of trans-affirming substance use harm reduction approaches.

## Introduction

There is a sizable body of evidence highlighting how trans and gender diverse young people (binary and non-binary, henceforth respectfully referred to using the heuristic umbrella term, ‘trans’) may be more likely to engage in substance use at earlier ages (Coulter et al., 2015), more often (Day et al., 2017; Strauss et al. 2020), and at higher intensities (Dermody et al., 2022; Hill et al., 2021), compared with their cisgender peers. Substance use during adolescence can lead to the development and entrenchment of chronic substance use-related harms and substance use disorders across the lifespan (Squeglia et al., 2009). Evidence from mainstream populations of young people has established that substance use often emerges in co-occurrence with mental ill-health during adolescence (Erskine et al., 2015; Solmi et al., 2022; McGrath et al., 2023), sharing many overlapping, compounding risk factors (Mewton et al., 2020; Birrell et al., 2020). This warrants particular attention because trans young people are also at increased risk of experiencing mental ill-health, such as depressive symptoms, anxiety symptoms, self-harm, and suicidality (Strauss et al., 2020; Hill et al., 2021; Bailey et al., 2024; Bailey et al., 2024b).

Despite this conceptual overlap, the literature regarding substance use among trans young people has largely been characterised by attempts to better quantify the burden of substance use among this population (Fahey et al., 2023; Connolly & Gilchrist 2020), with little research examining experiences of co-occurring substance use with mental ill-health despite their overlapping aetiologies (María et al., 2020; Reisner et al., 2016). Moreover, many relevant studies of co-occurring mental ill-health and substance use often focus on adult trans populations (Han et al., 2020), or sexuality diverse populations (Lea et al., 2014). The US-based Growing Up Today national cohort study of people aged 20–35 years (N = 12, 347) recruited from the general population found that trans participants had nearly three times increased odds of co-occurring depressive symptoms and alcohol use disorders compared with their cisgender peers (Felner et al., 2021). Conversely, a latent class analysis of co-occurring mental ill-health and substance use within a sample of trans and cisgender sexuality diverse youth aged 12 to 24 years (N = 392) seeking crisis services did not find an increased likelihood of co-occurring substance use and mental ill-health categories amongst trans young people compared with their cisgender sexuality diverse peers (Srivastava et al., 2020). It is important to note that the measurement of gender in previous studies, including Growing Up Today, typically utilise a dichotomised binary measurement of gender modality, that is, trans vs cisgender (Felner et al., 2021; Srivastava et al., 2020). Binary approaches to measuring gender may inadvertently exclude or misclassify non-binary trans youth. (Bailey et al., 2023).

There is also a paucity of research investigating potential correlates of co-occurring mental ill-health and substance use among trans young people. A recently published scoping review including 23 studies examining potential correlates of substance use among trans young people found that gender-based bullying, victimisation, and discrimination have been linked directly (Rowe et al., 2015; Watson et al., 2019; Pedro and Gorse 2023) and indirectly (Katz-Wise et al., 2021; Reisner et al., 2015) with elevated substance use within this group (Fahey et al., 2023). On the other hand, the mental health of trans young people has been linked to similar experiences. (Strauss et al., 2020; Hill et al., 2021; Bailey et al., 2024c)

In addressing co-occurring experiences of mental ill-health and substance use, it is important to note that there is difference between ‘substance use’ and ‘substance use harms and disorders’. Trans young people appear to use substances for pleasure-seeking and social group benefit (Freestone et al., 2022a; Freestone et al., 2022b). To address unintended and/or unwanted harms related to substance use, it is necessary to look at contextual characteristics of substance use, including substance use alone, or substance use for coping or self-medicative purposes. For example, a systematic review of solitary alcohol and cannabis use among adolescents in the United States general population found that earlier onset, heavier use, coping motives, negative emotions, and positive expectancies about use were significantly associated with solitary alcohol and cannabis use (Mason et al., 2020).Solitary substance use is prospectively associated with development of substance use disorders (Creswell et al., 2014; Tucker et al., 2006) and substance use-related harms such as feeling sick, getting into trouble, and missing school (Tucker et al., 2014). A recent online survey of trans young adults in Michigan, United States (N = 78; mean age = 21.3) found that solitary cannabis use was significantly associated with more frequent cannabis use (B = 17.73, *p* < 0.001), after controlling for key demographic factors and misuse of pain relievers, sedatives, and stimulants (Lee et al., 2023). Notwithstanding this, there is a paucity of research examining the contextual characteristics of substance use including solitary use and motives for use, among specifically trans young people. Addressing the contextual characteristics of substance use among trans youth is critical for elucidating why and how trans youth use substances. The context-dependent nature of substance use among trans youth is especially salient when considering how and why trans youth navigate substance use while also navigating co-occurring experiences of mental ill-health. This knowledge is requisite for better informing trans-affirming harm reduction efforts which account for the utility of substance use among trans youth and tailor supportive conversations to the unique context of substance use among trans youth.

Using data from the seminal Trans Pathways study of 859 trans young people in Australia (Strauss et al., 2020), the present study aimed to fill the research gaps identified above by focusing on three research aims: (1) to examine the association between mental ill-health and recent substance use among trans young people; (2) to examine the association between contextual characteristics of substance use (solitary use, coping motive for use) and mental ill-health outcomes; (3) to analyse associations between co-occurring mental ill-health and substance use outcomes with psychosocial stressors among trans young people.

## Methods

### Study Design and Population

The present study uses data from the Trans Pathways survey: a mixed-methods, cross-sectional survey conducted online from February 2016 to August 2016 open to trans young people aged 14–25 years in Australia. Eligibility criteria for young people included identifying as trans or gender diverse, being aged between 14 and 25 years at the time of the study, and currently resides in Australia. The Trans Pathways online questionnaire was promoted widely through social media (namely Facebook, Twitter, and Tumblr), trans and queer departments at universities, trans and queer support groups, parent support groups, trans peer-led safe spaces, various trans health and rights organisations throughout Australia, medical and mental health services, radio, and by word of mouth. The Trans Pathway study was approved by the University of Western Australia Human Research Ethics Committee (RA/4/1/7958) and the Clinical Evaluation and Research Committee of Youth Mental Health, North Metropolitan Health Service Mental Health, Department of Health, Western Australia. Further information on the Trans Pathways survey has been previously published. (Strauss et al., 2020)

### Predictive and Outcome Variables

#### Recent Substance Use

Participants were asked to indicate their substance use in the last 6 months, with 11 response options in total: tobacco, alcohol, cannabis, amphetamine type stimulants, inhalants, sedatives or sleeping pills, hallucinogens, opioids, other stimulants not for prescribed use, other (to be specified) or none (no substance use within the preceding six months). Participants checked all the options that applied. Each of these substance use items were dichotomised (1 = Yes, 0 = No) to indicate past six-month use of each eleven substance use item, respectively.

For the purposes of maintaining sufficient sample size to examine correlates of co-occurring mental ill-health and substance use outcomes, items assessing use of cannabis, amphetamine type stimulants, inhalants, sedatives or sleeping pills, hallucinogens, opioids, and other stimulants not for prescribed use were combined to indicate participants’ past six-month use of ‘other drugs’. Participants who responded ‘Yes’ to any of these constituent substances were coded as having recently used ‘other drugs’ whereas participants with ‘No’ responses to all of these constituent substances were coded as not having recently used ‘other drugs’.

Participants were also asked whether they had used alcohol or other drugs (apart from tobacco/cigarettes) when they were alone within the past six months (1 = ‘Yes’, 0 = ‘No’) and whether they had used substances to feel better about themselves and their life in the past six months (1 = ‘Yes’, 0 = ‘No’).

## Mental Health

### Depressive Disorder

For depressive symptoms, the Patient Health Questionnaire (PHQ-A) was used to assess depressive symptomatology through nine items assessing past two-week frequency of experiencing depressive symptoms (possible options ranging from 0 = Not at all to 3 = Nearly every day). Responses were summed for a total score of 0 to 27 (Johnson et al., 2002). A dichotomous categorical variable (yes/no) was computed using these sum scores, with > 15 indicating the presence of probable depressive disorder (Kroenke et al., 2001). Previous research shows the PHQ-9 possesses a high degree of reliability and validity when used as a screening tool for depression among adolescent populations, including in Australia. (Richardson et al., 2010)

### Anxiety Symptoms

The Generalised Anxiety Disorder Screening Tool (GAD-7) comprising seven items was used to assess past two-week anxiety symptoms (Spitzer et al., 2006) (e.g., “feeling nervous, anxious, or on edge’) with possible options ranging 0 (Not at all) to 3 (Nearly every day). Scores were summed for a possible range of 0 to 27 and a dichotomous categorical variable (yes/no) was computed using these sum scores with > 15 indicating the presence of probable anxiety disorder (Spitzer et al., 2006). Past research with adolescent populations shows the GAD-7 accurately reflects anxiety symptom severity and is associated with acceptable specificity and sensitivity for detecting clinically-significant anxiety symptoms. (Mossman et al., 2017)

### Self-Harm Thoughts / Behaviours, And Suicidal Thoughts/Attempts

Participants were also asked whether they had experienced self-harm thoughts (‘Have you ever wanted to harm yourself?’), self-harm behaviours (‘Have you ever harmed yourself?’), suicidal thoughts (‘Have you had series thoughts about ending your life?’), and suicide attempts (‘Have you tried to kill yourself or made a suicide attempt?’) within the last 12 months, prior to the last 12 months, never, or ‘prefer not to say’. The present paper examines responses reporting self-harm thoughts and behaviours, and suicidal thoughts and attempts, within the last 12 months only (four outcomes in total: self-harm thoughts, self-harm behaviours, suicidal thoughts, and suicide attempts).

## Interpersonal Stressors

Participants were asked 18 yes/no questions assessing exposure to different psychosocial and interpersonal experiences: ‘feeling rejected by peers’; ‘school, technical and further education (TAFE) or university issues, including missing school, grades dropping’ (issues with school, TAFE or university); ‘a lack of stable accommodation, including crisis accommodation, couch surfing, homelessness and risk of homelessness’ (unstable accommodation); ‘employment or unemployment issues’ (issues with employment); ‘body dysphoria (e.g., intense dislike of female or male anatomy)’; ‘feeling isolated and alone because of not knowing other trans people’; ‘feeling distressed or overwhelmed when trying to help friends or others with their mental health issues or problems’; ‘feeling isolated and alone because of unavailability of services’; ‘a significant loss’; ‘discrimination’; ‘a lack of family support’; ‘bullying, harassment, or verbal abuse (either in person or online); ‘sexual abuse within your family’, ‘physical abuse within your family’, ‘other abuse within your family (verbal, neglect, emotional); ‘sexual abuse outside of the family’; ‘physical abuse outside of the family’; and ‘abuse within an intimate relationship’. All 18 items comprised a specific section asking participants about a range of negative experiences that were hypothesised to be associated with poor mental health among trans young people (Strauss et al., 2020). All items were included in the present analyses. For the purposes of this study, a dichotomous categorical variable was used for each psychosocial, interpersonal experience (“Yes” = 1, “No” = 0) with participants responding ‘prefer not to say’ excluded.

### Analyses

Prevalence ratios were computed to assess prevalence of mental ill-health among trans young people, by recent substance use (past six-month use of smoking, alcohol, cannabis, amphetamine, inhalants, sedative, hallucinogens, opioid use, and other stimulants; and any ‘other drug’ use, which was defined as use of any of the aforementioned drugs excluding smoking and alcohol use). Multivariate logistic regression models were used to estimate adjusted odds ratios testing associations between these 10 recent substances use indicators and mental ill-health outcomes, controlling for age and gender (trans man, trans woman, and non-binary).

Prevalence ratios were also constructed to assess prevalence of mental ill-health among participants reporting recent substance use alone, and recent substance use to feel better about themselves and their life. Following this, multivariate logistic regression models were constructed to test associations between these contextual substance use characteristics and mental ill-health, controlling for age and gender (trans man, trans woman, non-binary).

Lastly, to examine correlates of co-occurring mental ill-health and substance use among this sample of trans youth, six co-occurring mental ill-health and substance use variables were computed: (1) past depression disorder and recent smoking; (2) probable depressive disorder and recent alcohol use; (3) probable depressive disorder and recent ‘other’ drug use; (4) probable anxiety disorder and recent smoking; (5) probable anxiety disorder and recent alcohol use; and (6) probable anxiety disorder and recent ‘other’ drug use. Multivariate logistic regression analyses were created to test associations between 18 interpersonal stressors and these six co-occurring mental ill-health and substance use outcomes, adjusting for age and gender.

Missing data for all recent substance use and recent substance use alone variables was under 5%, though there was 17% missing data for ‘using substances to feel better about oneself and life’. Between 20 to 24% data was missing for those 18 interpersonal stressors. To handle missing data, complete case analysis was used for all analysis, including only participants with no missing data on the corresponding variables. All analyses were conducted in R Studio Version 3.2.1. (Team RC 2021)

## Results

A total of 859 trans young people responded to the survey, of which 615 (71.6%) were aged 18 years or older and 244 (28.4%) were under 18 years of age. In terms of gender identity, 422 (52.4%) participants were non-binary, 255 (31.6%) participants were trans men, and 129 (16.0%) participants were trans women. Most participants were from Victoria (25%), New South Wales (20%), Queensland (17%), and Western Australia (16%).

### Co-occurring Depressive Symptoms and Recent Substance Use

Participants who reported recent smoking (aOR = 1.9, 95% CI: 1.3–3.0, *p* < 0.001), any ‘other drug’ use (aOR = 2.1, 1.4–3.2, *p* = 0.001), cannabis use (aOR = 1.8, 95% CI: 1.2–2.7, *p* = 0.01), or sedative use (aOR = 3.1, 95% CI: 1.7–5.9, *p* < 0.001) had significantly increased odds ratios for probable depressive disorder, relative to participants who did not use these drugs.

### Co-occurring Anxiety Symptoms and Recent Substance use

Participants who reported recent smoking (aOR = 1.9, 95% CI: 1.3–2.7, *p* < 0.001), any ‘other drug’ use (aOR = 1.8, 95% CI: 1.3–2.5, *p* < 0.001), cannabis use (aOR = 1.6, 95% CI: 1.1–2.2, *p* = 0.01), or sedative use (aOR = 2.7, 95% CI: 1.7–4.4, *p* < 0.001) also had significantly increased odds ratios for probable anxiety disorder, compared with non-users.

### Co-Occurring Self-Harm Thoughts and Behaviours with Recent Substance Use

Within this sample, participants who reported recent smoking (aOR = 0.5, 95% CI: 0.3–0.7, *p* < 0.001), any ‘other drug’ use (aOR = 0.4, 95% CI: 0.3–0.6, *p* < 0.001), cannabis use (aOR = 0.6, 95% CI: 0.4–0.8, *p* < 0.001), amphetamine use (aOR = 0.5, 95% CI: 0.2–0.9, *p* = 0.04), inhalants use (aOR = 0.3, 95% CI: 0.1–0.8, *p* = 0.03), sedative use (aOR + 0.4, 95% CI: 0.2–0.6, *p* < 0.001), or opioid use (aOR = 0.4, 95% CI: 0.2–0.9, *p* = 0.04) had significantly increased odds ratios for self-harm thoughts in the past 12 months, compared with participants who did not report recent alcohol or other drug use.

Among those who reported recent smoking (aOR = 0.4, 95% CI: 0.3–0.5, *p* < 0.001), any ‘other drug’ use (aOR = 0.4, 95% CI: 0.3–0.6, *p* < 0.001), cannabis use (aOR = 0.6, 95% CI: 0.4–0.8, *p* < 0.001), inhalants use (aOR = 0.3, 95% CI: 0.2–0.7, *p* < 0.001), sedatives use (aOR = 0.3, 95% CI: 0.2–0.5, *p* < 0.001), opioid use (aOR = 0.4, 95% CI: 0.2–0.8, *p* = 0.01) had significantly lower odds ratios self-harm behaviours in the past 12 months, compared with non-users.

### Co-Occurring Suicidal Thoughts and Attempts, with Recent Substance Use

Participants who reported recent smoking (aOR = 0.5, 95% CI: 0.4–0.7, *p* < 0.001), alcohol use (aOR = 0.7, 95% CI: 0.5–1.0, *p* = 0.03), any ‘other drug’ use (aOR = 0.4, 95% CI: 0.3–0.5, *p* < 0.001), cannabis use (aOR = 0.6, 95% CI: 0.4–0.8, *p* < 0.001), inhalants use (aOR = 0.4, 95% CI: 0.2–0.8, *p* = 0.01), or sedatives use (aOR = 0.4, 95% CI: 0.3–0.6, *p* < 0.001) reported significantly decreased odds of past 12-month suicidal thoughts compared with participants who did not report recent substance use.

Odds ratios were also significantly lower for suicide attempts in the past 12 months for participants who reported recent smoking (aOR = 0.3, 95% CI: 0.2–0.5, *p* < 0.001), any ‘other drug’ use (aOR = 0.4, 95% CI: 0.3–0.7, *p* < 0.001), cannabis use (aOR = 0.6, 95% CI: 0.4–1.0, *p* = 0.04), inhalants use (aOR = 0.4, 95% CI: 0.2–0.8, *p* = 0.01), sedatives use (aOR = 0.3, 95% CI: 0.2–0.5, *p* < 0.001), and other stimulants (aOR = 0.3, 95% CI: 0.1–0.9, *p* = 0.02), compared with non-users.

Full results are displayed below in Table 1.

**Table 1:** Prevalence of mental ill-health among trans young people by past six-month substance use

	Probable depressive order	Probable anxiety disorder	Past 12-month self-harm thoughts	Past 12-month self-harm	Past 12-month suicide thoughts	Past 12-month suicide attempt
	N (%) aOR, 95% CI *p*	N (%) aOR, 95% CI *p*	N (%) aOR, 95% CI *p*	N (%) aOR, 95% CI *p*	N (%) aOR, 95% CI *p*	N (%) aOR, 95% CI *p*
Cigarette smoking	178 (82.0) 1.9, 1.3–3.0 < *0.001*	154 (71.0) 1.9, 1.3–2.7 < *0.001*	53 (24.3) 0.5, 0.3–0.7 < *0.001*	92 (42.2) 0.4, 0.3–0.5 < *0.001*	94 (43.1) 0.5, 0.4–0.7 < *0.001*	165 (75.5) 0.3, 0.2–0.5 < *0.001*
Alcohol	385 (76.1) 1.2, 0.8–1.9 *0.34*	307 (61.3) 1.0, 0.7–1.5 *0.93*	169 (33.5) 0.8, 0.5–1.2 *0.30*	288 (56.8) 0.8, 0.6–1.2 *0.36*	255 (50.4) 0.7, 0.5–1.0 *0.03*	427 (84.2) 0.6, 0.4–1.1 *0.11*
Any ‘other drug’ use^1^	243 (82.7) 2.1, 1.4–3.2 *0.001*	198 (68.8) 1.8, 1.3–2.5 < *0.001*	70 (23.9) 0.4, 0.3–0.6 < *0.001*	136 (46.4) 0.4, 0.3–0.6 < *0.001*	117 (39.8) 0.4, 0.3–0.5 < *0.001*	233 (79.3) 0.4, 0.3–0.7 < *0.001*
Cannabis	176 (82.2) 1.8, 1.2–2.7 *0.01*	146 (68.9) 1.6, 1.1–2.2 *0.01*	55 (25.8) 0.6, 0.4–0.8 < *0.001*	105 (49.1) 0.6, 0.4–0.8 < *0.001*	92 (43.0) 0.6, 0.4–0.8 < *0.001*	174 (81.3) 0.6, 0.4–1.0 *0.04*
Amphetamine	40 (78.4) 1.3, 0.7–2.9 *0.46*	36 (72.0) 1.8, 0.9–3.6 *0.09*	12 (23.5) 0.5, 0.2–0.9 *0.04*	26 (51.0) 0.7, 0.4–1.2 *0.20*	25 (49.0) 0.8, 0.4–1.4 *0.37*	43 (84.3) 0.7, 0.3–1.8 *0.47*
Inhalants	28 (80.0) 1.1, 0.5–2.9 *0.79*	26 (74.3) 1.7, 0.8–3.9 *0.20*	6 (17.1) 0.3, 0.1–0.8 *0.03*	12 (34.3) 0.3, 0.2–0.7 < *0.001*	11 (31.4) 0.4, 0.2–0.8 *0.01*	24 (68.6) 0.4, 0.2–0.8 *0.01*
Sedative	115 (88.5) 3.1, 1.7–5.9 < *0.001*	98 (77.2) 2.7, 1.7–4.4 < *0.001*	24 (18.5) 0.4, 0.2–0.6 < *0.001*	47 (36.2) 0.3, 0.2–0.5 < *0.001*	44 (33.8) 0.4, 0.3–0.6 < *0.001*	93 (71.5) 0.3, 0.2–0.5 < *0.001*
Hallucinogen	29 (78.4) 1.1, 0.5–2.6 *0.87*	24 (66.7) 1.3, 0.6–2.7 *0.55i*	11 (29.7) 0.7, 0.3–1.4 *0.30*	19 (51.4) 0.6, 0.3–1.3 *0.21*	18 (48.6) 0.8, 0.4–1.6 *0.48*	32 (86.5) 0.8, 0.3–2.5 *0.72*
Opioid use	36 (81.8) 1.7, 0.8–4.2 *0.24*	31 (72.1) 2.0, 1.0–4.5 *0.06*	9 (20.5) 0.4, 0.2–0.9 *0.04*	18 (40.9) 0.4, 0.2–0.8 *0.01*	17 (38.6) 0.5, 0.3–1.0 *0.06*	39 (88.6) 1.1, 0.4–3.2 *0.88*
Other stimulants	17 (73.9) 0.7, 0.3–2.1 *0.53*	17 (73.9) 1.9, 0.7–5.8 *0.23*	3 (13) 0.3, 0.1–1.1 *0.10*	5 (22.7) 0.3, 0.1–0.7 *0.01*	10 (43.5) 0.7, 0.3–1.6 *0.38*	16 (69.6) 0.3, 0.1–0.9 *0.02*

^1^The ‘other drug’ use item is a composite variable indicating those who responded ‘yes’ to any of the following drugs: cannabis, amphetamine, inhalants, sedatives, hallucinogens, opioid use, and other stimulants

### Experiences of Mental Ill-Health Among Participants Who Have Recently Used Substances While they Were Alone or to Feel Better About Themselves

Participants who reported recently using substances while they were alone had lower odds ratios for probable depressive disorder (aOR = 0.6, 95% CI: 0.4–0.9, *p* = 0.02) and probable anxiety disorder (aOR = 0.6, 95% CI: 0.5–0.9, *p* = 0.01), compared with those who did not report recently using substances while alone. On the other hand, participants who reported recently using substances while they were alone were at increased risk of self-harm thoughts (aOR + 1.6, 95% CI: 1.2–2.3, *p* < 0.001), past 12-month self-harm attempt/s (aOR = 2.4, 95% CI: 1.7–3.3, *p* < 0.001), past 12-month suicidal thoughts (aOR = 1.8, 95% CI: 1.3–2.5, *p* < 0.001), and past 12-month suicide attempt/s (aOR = 2.8, 95% CI: 1.8–4.5, *p* < 0.001), compared with those who did not report recently using substances while alone.

Participants who reported recently using substances to feel better about themselves and their lives had significantly increased odd ratios for self-harm thoughts in the past 12 months (aOR = 2.8, 95% CI: 1.5–2.9, *p* < 0.001), self-harm attempt/s in the past 12 months (aOR = 2.0, 95% CI: 1.2–3.2, *p* = 0.01), suicidal thoughts in the past 12 months (aOR = 1.8, 95% CI: 1.1–2.9, *p* = 0.02), compared with those who did not report recently substance use in order to feel better about themselves and their lives.

Full results are shown below in Table 2.

**Table 2:** Associations between mental ill–health symptoms and contextual characteristics of substance use

	Probable depressive order	Probable anxiety disorder	Past 12-month self-harm thoughts	Past 12-month self-harm attempt	Past 12-month suicide thoughts	Past 12-month suicide attempt
	N (%) aOR, 95% CI *p*	N (%) aOR, 95% CI *p*	N (%) aOR, 95% CI *p*	N (%) aOR, 95% CI *p*	N (%) aOR, 95% CI *p*	N (%) aOR, 95% CI *p*
Substance use alone	304 (74.5) 0.6, 0.4–0.9 *0.02*	240 (59.6) 0.6, 0.5–0.9 *0.01*	151 (36.8) 1.6, 1.2–2.3 < *0.001*	261 (63.3) 2.4, 1.7–3.3 < *0.001*	235 (57.2) 1.8, 1.3–2.5 < *0.001*	367 (89.1) 2.8, 1.8–4.5 < *0.001*
Using substances to feel better about oneself and life	206 (85.5) 0.9, 0.5–1.8 *0.80*	170 (71.1) 1.1, 0.6–1.7 *0.84*	56 (23.1) 2.8, 1.5–2.9 < *0.001*	105 (43.4) 2.0, 1.2–3.2 *0.01*	99 (40.9) 1.8, 1.1–2.9 *0.02*	190 (78.5) 1.6, 0.9, 2.6 *0.09*

### Correlates of Co-occurring Depression/Anxiety and Substance Use

Nearly all correlates examined were significantly associated with one or more co-occurring depression/anxiety and substance use outcomes. Full results are displayed in Table 3 below. In brief, issues with school, TAFE, or university produced the largest effect sizes associated with co-occurring depression/anxiety and substance use, followed by unstable accommodation, and experiences of intimate partner abuse.

**Table 3:** Determinants of co-occurring depression, anxiety, and substance use

	Probable depressive disorder and recent smoking	Probable depressive disorder and recent alcohol use	Probable depressive disorder and recent ‘other’ drug use^1^	Probable anxiety disorder and recent smoking	Probable anxiety disorder and recent alcohol use	Probable anxiety disorder and recent ‘other’ drug use
	aOR, 95% CI *p*	aOR, 95% CI *p*	aOR, 95% CI *p*	aOR, 95% CI *p*	aOR, 95% CI *p*	aOR, 95% CI *p*
Peer rejection	1.6, 1.0–2.6 *0.03*	1.2, 0.8–1.7 *0.34*	1.4, 0.9–2.1 *0.10*	1.9, 1.2–3.0 *0.01*	1.5, 1.0–2.1 *0.04*	1.8, 1.2–2.7 *0.01*
Issues with school	2.9, 1.7–5.3 < *0.001*	3.1, 2.1–4.7 < *0.001*	3.1, 1.9–5.2 < *0.001*	2.3, 1.4–3.9 < *0.001*	3.0, 2.0–4.6 < *0.001*	2.7, 1.7–4.5 < *0.001*
Issues with accommodation	3.3, 2.2–5.0 < *0.001*	1.6, 1.1–2.4 *0.02*	2.8, 1.9–4.2 < *0.001*	3.7, 2.4–5.7 < *0.001*	1.8, 1.2–2.8 < *0.001*	2.5, 1.7–3.8 < *0.001*
Issues with employment	2.0, 1.4–3.0 < *0.001*	1.9, 1.3–2.7 < *0.001*	1.9, 1.3–2.8 < *0.001*	2.0, 1.3–3.0 < *0.001*	1.8, 1.3–2.6 < *0.001*	1.8, 1.2–2.6 < *0.001*
Body dysphoria	1.6, 0.7–4.1 *0.27*	2.0, 1.0–4.0 *0.05*	1.7, 0.8–3.8 *0.16*	1.9, 0.8–5.1 *0.18*	0.9, 0.5–1.8 *0.85*	1.4, 0.7–3.1 *0.35*
Isolated and alone due to not knowing other trans people	1.7, 1.1–2.5 *0.01*	1.6, 1.1–2.2 *0.01*	1.5, 1.0–2.1 *0.03*	1.7, 1.2–2.6 *0.01*	1.1, 0.8–1.5 *0.66*	1.3, 0.9–1.9 *0.13*
Overwhelmed from helping others	1.8, 1.2–2.7 *0.01*	1.4, 1.0–2.0 *0.06*	1.8, 1.2–2.6 < *0.001*	1.4, 0.9–2.1 *0.11*	1.0, 0.7–1.5 *0.88*	1.7, 1.2–2.5 *0.01*
Isolated from services	1.5, 1.0–2.2 *0.03*	1.3, 0.9–1.8 *0.11*	1.5, 1.1–2.2 *0.02*	1.5, 1.0–2.2 *0.04*	1.1, 0.8–1.5 *0.73*	1.3, 0.9–1.8 *0.15*
Significant loss	2.4, 1.7–3.6 < *0.001*	1.4, 1.0–2.0 *0.04*	2.0, 1.4–2.8 < *0.001*	2.3, 1.6–3.4 < *0.001*	1.3, 1.0–1.9 *0.09*	2.0, 1.4–2.8 < *0.001*
Lack of family support	1.7, 1.1–2.6 *0.01*	1.3, 0.9–1.8 *0.16*	1.6, 1.1–2.4 *0.01*	1.9, 1.3–2.9 < *0.001*	1.4, 1.0–1.9 *0.08*	1.5, 1.1–2.2 *0.02*
Discrimination	2.4, 1.6–3.9 < *0.001*	1.9, 1.3–2.7 < *0.001*	2.4, 1.6–3.7 < *0.001*	2.6, 1.7–4.2 < *0.001*	1.7, 1.2–2.5 < *0.001*	2.0, 1.4–3.0 < *0.001*
Bullying	1.6, 1.0–2.6 *0.03*	1.2, 0.8–1.7 *0.34*	1.4, 0.9–2.1 *0.10*	1.9, 1.2–3.0 *0.01*	1.5, 1.0–2.1 *0.04*	1.8, 1.2–2.7 *0.01*
Family sexual abuse	1.6, 0.8–2.9 *0.16*	0.9, 0.5–1.6 *0.72*	1.4, 0.8–2.5 *0.29*	1.5, 0.8–2.8 *0.17*	0.9, 0.5–1.6 *0.67*	0.9, 0.5–1.7 *0.84*
Family physical abuse	1.8, 1.2–2.7 < *0.001*	1.3, 0.9–1.8 *0.25*	1.7, 1.2–2.5 < *0.001*	2.3, 1.5–3.4 < *0.001*	1.5, 1.0–2.2 *0.05*	1.8, 1.2–2.6 < *0.001*
Family other abuse	1.9, 1.3–2.8 < *0.001*	1.7, 1.2–2.4 < *0.001*	2.0, 1.4–2.8 < *0.001*	1.9, 1.3–2.8 < *0.001*	1.6, 1.2–2.3 < *0.001*	1.7, 1.2–2.4 < *0.001*
Sexual abuse outside family	1.6, 0.8–2.9 *0.16*	0.9, 0.5–1.6 *0.72*	1.4, 0.8–2.5 *0.29*	1.5, 0.8–2.8 *0.17*	0.9, 0.5–1.6 *0.67*	0.9, 0.5–1.7 *0.84*
Physical abuse outside family	2.5, 1.6–3.9 < *0.001*	1.1, 0.7–1.8 *0.59*	2.3, 1.5–3.5 < *0.001*	2.6, 1.6–4.1 < *0.001*	1.4, 0.9–2.2 *0.17*	2.4, 1.5–3.7 < *0.001*
Intimate partner abuse	2.9, 2.0–4.3 < *0.001*	1.8, 1.3–2.6 < *0.001*	2.2, 1.6–3.2 < *0.001*	3.0, 2.0–4.4 < *0.001*	1.7, 1.2–2.5 < *0.001*	2.0, 1.4–2.9 < *0.001*

^1^The ‘other drug’ use item is a composite variable indicating those who responded ‘yes’ to any of the following drugs: cannabis, amphetamine, inhalants, sedatives, hallucinogens, opioid use, and other stimulants

## Discussion

This paper contributes a number of novel insights into the frequency, correlates, and contextual characteristics of experiences of co-occurring mental ill-health and substance use among trans young people. Through systematically analysing and contextualising experiences of co-occurring mental ill-health and substance use, this paper aims to inform a more nuanced basis for harm-reduction approaches and prevention of substance use among trans young people.

### Interconnectedness of Mental Health and Substance Use Among Trans Young People

Our findings suggest trans young people are twice as likely to report psychiatric comorbidity alongside illicit drug use. Between 69 to 83% of our sample who reported recent illicit drug use also reported depression and/or anxiety whereas in the mainstream population of young people, just over one third (36%) of young people with self-reported mental ill-health engaged in illicit drug use within the past 12 months. (Institute and of Health and Welfare.Alcohol,tobacco and other drugs 2021 [Available from:https: www.aihw.gov.au, reports, children-youth, alcohol-tobacco-and-other-drugs. 2021) Given that data for the present study was collected in 2016, further research is urgently required to monitor illicit drug use and co-occurring mental ill-health among trans young people as these trends likely do not converge with the mainstream population of young people who report decreasing rates of illicit drug use in recent years. (Institute and of Health and Welfare.Alcohol,tobacco and other drugs 2021 [Available from:https: www.aihw.gov.au, reports, children-youth, alcohol-tobacco-and-other-drugs. 2021)

Our findings suggest that there are distinct patterns of co-occurring experiences mental ill-health and substance use, particularly trans young people with depression and anxiety, co-using sedatives or smoking cigarettes. Co-occurring experiences of depression and anxiety with sedatives and smoking, in particular, may expose trans young people to additional potential harms related to potential medication interactions (NSW Department of Health 2007). Where trans young people may be co-using anti-depressants alongside opiates or sedatives (such as benzodiazepines), the likelihood of overdose is significantly increased, and the therapeutic utility of the prescribed medications may be reduced (NSW Department of Health 2007; Marel et al., 2022). In 2021–2022, 25% of young people aged 12–24 years in Australia had dispensed one or more mental health-related medications (Australian Institute of Health and Welfare 2023a), with longitudinal trends suggesting the number of mental health-related prescriptions is increasing across the Australian population (Institute and of Health and Welfare 2023b). Recent estimates suggest over half of trans young people are currently diagnosed with depression and anxiety, with around two-thirds of these people receiving treatment (Hill et al., 2021). To inform future health promotion efforts, more research is required assessing types and classes of medications (including but not limited to psychotropic medications) to allow for more systematic assessment of potential risks and harms associated with substance use among trans young people.

### The Case for Trans-Affirming Models of Harm Reduction

We suggest harm reduction practitioners should also consider our findings that substance use was associated with a reduction in risk of self-harm and suicide outcomes. There is limited corroborating these findings, however, a previous study using data from the US-based 2017 Youth Risk Behavior Survey (N = 17,853) found that exposure to various forms of adversity (e.g., school-based bullying), past 30-day cigarette, alcohol, and marijuana use was associated with significantly increased odds of suicide attempt/s and suicide attempt/s requiring treatment among LGBTQA + young people (aORs ranging 1.19 to 2.65, depending on substance use and adjusting for gender identity, age, race, and sexuality) (Jackman et al., 2021). However, this sample comprised ~ 3% trans participants and ~ 97% cisgender participants, thus limiting the generalisability of these findings to trans young people. Our findings add a further layer of complexity to the literature which has traditionally characterised self-harm and suicidality in isolation from substance use (Newcomb et al., 2020; White et al., 2023) under the assumption that both are psychological sequelae from gender minority stressors such as gender-based discrimination, bullying, and victimisation (Garthe et al., 2022). Previous analyses of Trans Pathways data has shown that a significant proportion of trans young people use substances for social, altruistic, and community-seeking reasons (Freestone et al., 2022a; Freestone et al., 2022b). Although the literature on cisgender populations suggests that self-harm and suicidality may be reciprocally (Zhang and Wu 2014) associated with increased substance use through social withdrawal, isolation, and breakdown of social bonds (Pompili et al., 2010), our study suggests this may not necessarily be the case for trans young people, who may potentially experience positive and meaningful affective experiences from using substances in certain social, altruistic, and community-seeking contexts. Continued harm-reduction, strengths-based approaches toward understanding substance use among trans young people are warranted, with particular attention on the embodied contexts and opportunity costs of substance use within communities of trans young people.

### The Importance but Not Centrality of Solitary Use of Alcohol and Other Drugs

Our findings indicated that, of the trans young people who reported recently using substances while they were alone or using substances to feel better about themselves and their life, nearly four in five had recently attempted suicide in the preceding 12 months. These findings endorse a non-judgemental harm reduction approach which centrally reflects on *why* a trans young person is using substances with the aim of promoting help-seeking efficacy, improving general and trans-specific coping skills, and cultivating connections with other young people, including trans communities. Though solitary substance use may be a useful litmus test of substance use harms under certain circumstances, our findings underscore the value of in-depth conversations with trans young people over and above use of brief survey or screening items. Trans youth likely use substances for more than one reason which vary in weight according to temporal and social contextual factors. Indeed, our findings make the case for trans-affirming harm reduction, characterised principally by the facilitation of trans-affirming conversations exploring the affective function of substance use unique for each person. Critically, clinicians and harm reduction practitioners should strive to facilitate these discussions in a balanced way with an informed willingness to acknowledge the positive and/or negative affects attached to these highly individual substance use experiences. Trans youth engaging in solitary substance use within the overarching context of substance use for coping motives may benefit from trans-affirming, supportive conversations around alternate coping skills which may be helpful. These conversations should avoid pathologising solitary substance use and strive to retain positive aspects of substance use outside of coping motives where possible, such as social connection and community participation.

### The Promise of Future School-Based Harm Reduction Efforts

This study also found an increased likelihood of all six co-occurring mental ill-health and substance use outcomes was observed among participants who reported recently experiencing issues with school, accommodation or employment, discrimination, other family abuse, and intimate partner abuse. These findings provide support for other studies indicating the importance of these direct and indirect distal gender minority stressors in determining trans health (Strauss et al., 2020; Tan et al., 2019). Although these psychosocial experiences span many settings—including schools, workplaces, home, and community—recent research has highlighted the role played by educational settings in in mediating mental health outcomes among trans young people (Green et al., 2018; Marx and Kettrey 2016; Day et al., 2020; Ancheta et al., 2021). Schools are well-placed to affirm and support trans young people through delivering trans-affirming peer support, counselling support, and referral pathways, as well as trans-inclusive facilities, curriculum, and administration (US Department of Health and Human Services. LGBTQ Inclusivity in Schools: A self-assessment tool 2020). Furthermore, schools may represent an appropriate site for delivering substance use prevention programs which promote agency over one’s substance use decisions and develop coping skills and harm minimisation strategies (Newton et al., 2022). Resources for substance use harm reduction tailored to trans young people could also be feasibly delivered through schools.

### Limitations

This study possessed many methodological strengths, including recruitment of a large sample of trans young people and comprehensive measures of different types of substance use. There were were also a number of limitations. Firstly, substance use was measured using past six-month exposure without capturing levels of intensities or frequencies, thereby limiting inference into potential harms related to substance use. This limitation was partially mitigated through the inclusion of items relating to the contextual characteristics of substance use episodes, specifically substance use while alone and using substances to feel better about oneself and one’s life. Secondly, the cross-sectional nature of this study constrains our ability to make causal claims regarding different aspects of mental ill-health and substance use among trans young people. Further longitudinal research is required to investigate the causal robustness of these associations. Thirdly, due to low endorsement of certain items, we were unable to examine correlates of co-occurring experiences of self-harm, suicidality, and substance use. Lastly, this study was conducted in 2016 hence participants’ data may have reduced generalisability to contemporary cohorts of trans youth.

## Conclusion

In summation, trans young people using substances, and particularly cigarettes, sedatives, and opioids, often do so with co-occurring experiences of depression and anxiety. However, it is possible that limited substance use in more “social” contexts may confer benefits in terms of significant self-harm and suicide prevention. We suggest these findings provide an evidence basis for continued research working directly with trans young people to conceptualise more nuanced and precise parameters of harm reduction approaches.
